# Supplementary material for: Aotaphenazine, a rare hydrophenazine, targets topoisomerase II with anticancer efficacy: In silico to in vitro evidence
Source: PLoS One. 2025 Dec 5;20(12):e0338135. doi: 10.1371/journal.pone.0338135 (PMC12680189; doi:10.1371/journal.pone.0338135)
Supplement: S1 File — (PDF) [file pone.0338135.s001.pdf]

Supporting Information

Aotaphenazine, a Rare Hydrophenazine, Targets Topoisomerase II with Anticancer Efficacy: In Silico to In Vitro Evidence

Ahmed M. Metwaly[a] *, Ibrahim H. Eissa[b]*, Wael M. Afifi[a, c], Eslam B. Elkaeed[d], Aisha A. Alsfouk[e], Ibrahim M. Ibrahim[f], Mohamed S. Abdelfattah[g]

[a] Pharmacognosy and Medicinal Plants Department, Faculty of Pharmacy (Boys), Al-Azhar University, Cairo 11884, Egypt

[b] Pharmaceutical Medicinal Chemistry & Drug Design Department, Faculty of Pharmacy (Boys), Al-Azhar University, Cairo11884, Egypt.

[c] Department of Pharmacognosy, Faculty of Pharmacy, Sinai University—Kantara Branch, Ismailia 41636, Egypt

[d] Department of Pharmaceutical Sciences, College of Pharmacy, AlMaarefa University, Riyadh 13713, Saudi Arabia.

[e] Department of Pharmaceutical Sciences, College of Pharmacy, Princess Nourah bint Abdulrahman University, P.O. Box 84428, Riyadh 11671, Saudi Arabia;

[f] Biophysics Department, Faculty of Science, Cairo University. Giza 12613, Egypt

[g] Chemistry Department, Faculty of Science, Helwan University, Cairo, Egypt.

***Corresponding authors:**

Ahmed M. Metwaly **Email:** ametwaly@azhar.edu.eg

Ibrahim H. Eissa **Email:** Ibrahimeissa@azhar.edu.eg

Content

**1**	**Molecular Docking**
**2**	**MD Simulation**
**3**	**MM-GBSA**
**4**	**ProLIF**
**5**	**PLIP**
**6**	***In vitro* Topo II Inhibition Assay**
**7**	**Cytotoxicity**
**8**	**Flow Cytometry**

- **1. Molecular Docking studies**

Protein Preparation:

The crystal structure of DNA–Topo II complex (PDB ID: 3QX3) was obtained from Protein Data Bank (https://www.rcsb.org). At first, the crystal structure of the protein complexed with (Dox) the co-crystallized ligand was prepared by removing crystallographic water molecules. Only one chain was retained besides the co-crystallized ligand. The selected protein chain was protonated using the following setting. The used electrostatic functional form was GB/VI with a distance cut-off of 15 Å. The used value of the dielectric constant was 2 with an 80 dielectric constant of the used solvent. The used Van der Waals functional form was 800R3 with a distance cut-off of 10 Å. Then, the energy of the protein chain was minimized using Hamiltonian AM1 implanted in Molecular Operating Environment (MOE 2019 and MMFF94x (Merck molecular force field) for structural optimization. Next, the active site of the target protein was defined for ligand docking and redocking (in case of validation of docking protocol). The active site of the protein was identified as the residues that fall within the 5 Å distance from the perimeter of the co-crystallized ligand.

Ligand Preparation: 2D structures of aotaphenazine and the standard compound, Dox were drawn using ChemBioDraw Ultra 14.0 and saved in MDL-SD file format. The 3D structures of the ligands were protonated, and the structures were optimized by energy minimization using MM2 force-field and 10000 iteration steps of 2 fs. The conformationally optimized ligands were used for docking studies.

Docking Setup and Validation of Docking Protocol: The protein-ligand docking studies were carried out using MOE version 2019. Validation of the docking protocol was carried out by redocking the co-crystallized reference ligand (Dox) against the isolated pocket of TOPO-

2. The docking protocol was validated by comparing the heavy atoms RMSD value of the re-docked ligand pose with the corresponding co-crystallized reference ligand structure.

The docking setup for aotaphenazine was established according to the protocol followed in the validation step. For each docking run, 30 docked solutions were generated using ASE for scoring function and rigid receptor for refinement. The pose with ideal binding mode was selected for further investigations. The docking results were visualized using Discovery Studio (DS) 4.0. Analysis of the docking results was carried out by comparing the interactions and docking score obtained for the docked ligands with that of the re-docked reference molecule (Dox).

2. Molecular Dynamic (MD) Simulation:

In this work, we employed unbiased molecular dynamics (MD) simulations to assess the stability of the Topo_DNA-Aotaphenazine complex. The simulations were executed using the GROMACS 2021 software package for a total duration of 400 nanoseconds. Initial configurations and topologies were generated utilizing the CHARMM-GUI solution builder module. The complex was embedded in a cubic simulation box with a side length of 13 nm. Solvation was achieved using the transferable intermolecular potential 3 points (TIP3P) water model, supplemented by a 1 nm solvent padding layer. To maintain system neutrality, NaCl ions were introduced at a concentration of 0.154 M. The CHARMM36m force field was utilized to describe the Topo protein, DNA molecules, TIP3P water model, and ions, while the Aotaphenazine molecule was parameterized using the CGenFF program.

The system was modeled with periodic boundary conditions (PBC) applied in all three dimensions. System preparation involved an energy minimization step (maximum force threshold of 10 kJ/(mol.nm), 200,000 steps maximum) to resolve atomic clashes. Two stages of equilibration were then initiated. Firstly, we used the NVT ensemble with velocity rescaling thermostat to maintain the temperature at 310 K. Subsequently, we employed the NPT ensemble with Berendsen pressure coupling algorithm to maintain the pressure at 1 atm and used velocity rescaling to maintain the temperature at 310 K. Production MD (400 ns) employed the NPT ensemble with Nose-Hoover temperature control set at 310 K and Parrinello-Rahman pressure coupling set at 1 atm. LINear Constraint Solver (LINCS) constrained hydrogen bonds in all of the steps to be able to use a time step of 2 femtoseconds , and electrostatics were treated with the Particle Mesh Ewald (PME) method with 1.2 nm cutoff . Equations of motion were integrated using the leap-frog algorithm (1 fs timestep for equilibration, 2 fs for production). Production run frames were saved every 0.1 ns, yielding 4,000 frames.

Following trajectory recentering (gmx trjconv) to maintain protein integrity within the simulation box, we performed comprehensive trajectory analyses using VMD TK scripts (10). Calculations included Root Mean Square Deviation (RMSD) of both Topo and Aotaphenazine to assess conformational stability. Additional structural analyses encompassed Root Mean Square Fluctuation (RMSF) to identify flexible regions, Radius of Gyration (RoG) to quantify compactness, and Solvent Accessible Surface Area (SASA) to measure solvent exposure. We closely monitored hydrogen bonding interactions between the ligand and Topo, and determined the time evolution of the distance between their respective centers of mass.

- **3. Molecular Mechanics-Generalized Born Surface Area (MM-GBSA)**

MM-GBSA implemented in gmx_MMPBSA program to determine the ligand's binding energy. To elucidate the contributions of individual amino acid residues or nucleic acid bases to the binding of the Aotaphenazine, we conducted a decomposition analysis within a 1 nm radius of the ligand. Calculation parameters included an ionic strength of 0.154 M, an igb solvation value of 5, and internal and external dielectric constants of 1.0 and 78.5, respectively. The theoretical basis of the MM-GBSA method can be expressed as follows (Equation 1):

$$\Delta G = \langle G_{complex} - (G_{receptor} + G_{ligand}) \rangle \qquad \text{Equation 1}$$

Where < > represents the average of the enclosed free energies of complex, receptor, and ligand over the frames used in the calculation. In our approach, we used the whole trajectory (a total of 4000 frames). Different energy terms can be calculated according to Equations 2 to 6 as follows:

$$\Delta G_{binding} = \Delta H - T\Delta S \qquad \text{Equation 2}$$

$$\Delta H = \Delta E_{gas} + \Delta E_{sol} \qquad \text{Equation 3}$$

$$\Delta E_{gas} = \Delta E_{ele} + \Delta E_{vdW} \qquad \text{Equation 4}$$

$$\Delta E_{solv} = E_{GB} + E_{SA} \qquad \text{Equation 5}$$

$$E_{SA} = \gamma.SASA \qquad \text{Equation 6}$$

Where: ΔH is the enthalpy which can be calculated from gas-phase energy (E_{gas}) and solvation-free energy (E_{sol}). -TΔS is the entropy contribution to the free binding energy. E_{gas} is composed of electrostatic and van der Waals terms; E_{ele}, E_{vdW}, respectively. E_{sol} can be calculated from the polar solvation energy (E_{GB}) and nonpolar solvation energy (E_{SA}) which is estimated from the solvent-accessible surface area.

- **4. Protein-Ligand Interaction Fingerprint (ProLIF) Analysis**

A comprehensive residue-specific interaction analysis was conducted across all 40,000 frames of the molecular dynamics trajectory (sampled at 10 ps intervals) using the ProLIF Python package (v1.0.0). The analysis employed the following rigorous criteria:

- **Interaction Detection Parameters**:
  - *Hydrogen bonds*: Required a donor-acceptor distance < 3.5 Å and a bond angle > 120°, with potential donors/acceptors identified according to CHARMM36 topology rules
  - *Hydrophobic contacts*: Defined as nonpolar atom pairs (C-C, C-S) within 4.5 Å without angle restrictions
  - *π-interactions*: Included both π-π stacking (aromatic ring centroids ≤5.5 Å with face-to-face or offset orientations) and cation-π interactions (charged groups ≤5.0 Å from ring centroids)
  - *Ionic pairs*: Oppositely charged residues (Asp/Glu vs. Arg/Lys/His) within 4.0 Å
- **Statistical Evaluation**:
  Interaction persistence was quantified using the metric: Persistence (%) = (Number of frames with interaction/Total analyzed frames) × 100. This statistical measure was calculated across all 20,000 trajectory frames (sampled at 10 ps intervals) to determine the thermodynamic stability of each ligand-residue contact. Only interactions demonstrating >70% persistence (equivalent to >14,000 observable frames) were considered biologically relevant, corresponding to $p < 0.01$ in a binomial distribution test against random binding events. The resulting persistence values were mapped to specific binding pockets, revealing three distinct interaction regimes: transient (<30%), intermediate (30-70%), and stable (>70%) contact residues.

- **5. Protein-Ligand Interaction Profiler (PLIP) Analysis**

The molecular dynamics trajectory was systematically analyzed using the PLIP through an optimized four-stage workflow: First, conformational clustering was performed using the TTClust algorithm with a backbone RMSD cutoff of 2.0 Å (CHARMM36 force field parameters), which identified three dominant metastable states collectively representing 82±4% of the simulation time. From these clusters, centroid frames were extracted as representative structures after verifying their structural similarity to cluster members (average intra-cluster RMSD 1.2±0.3 Å). Each representative complex was then submitted to the PLIP webserver (https://plip-tool.biotec.tu-dresden.de) for comprehensive interaction mapping, which generated: (i) detailed 2D interaction diagrams annotating hydrogen bonds (distance ≤3.5 Å, angle ≥120°), π-stacking (interplanar distance ≤5.5 Å), and salt bridges (≤4.0 Å); and (ii) PyMOL-compatible 3D visualization files (.pse format, PyMOL v2.5.2) with customized coloring schemes for different interaction types. Interaction persistence metrics were calculated across clusters as (ninteractions/ntotal clusters)×100%, revealing key residues with 100% interaction consistency. All analysis files, including PLIP configuration templates (XML format).

7. Cytotoxicity assay

Human lung fibroblast cell line (WI-38) , Human amnion (WISH), Hepatocellular carcinoma (HePG-2) , Epitheliod Carcinoma (Hela) , Human prostate cancer (PC3), Mammary gland (MCF-7), Colorectal adenocarcinoma (Caco-2), Colorectal carcinoma (HCT-116), and Breast cancer (MDA-MB-231). The cell line were obtained from ATCC via Holding company for biological products and vaccines (VACSERA), Cairo, Egypt.

Doxorubicin was used as a standard anticancer drug for comparison.

The reagents RPMI-1640 medium , MTT and DMSO (sigma co., St. Louis, USA), Fetal Bovine serum (GIBCO, UK) .

The cell lines mentioned above were used to determine the inhibitory effects of compounds on cell growth using the MTT assay. This colorimetric assay is based on the conversion of the yellow tetrazolium bromide (MTT) to a purple formazan derivative by mitochondrial succinate dehydrogenase in viable cells. Cell lines were cultured in RPMI-1640 medium with 10% fetal bovine serum. Antibiotics added were 100 units/ml penicillin and 100µg/ml streptomycin at 37 C in a 5% Co_2 incubator. The cell lines were seedes in a 96-well plate at a density of $1.0x10^4$ cells/well. at 37 C for 48 h under 5% Co_2. After incubation the cells were treated with different concentration of compounds and incubated for 24 h. After 24 h of drug treatment, 20 µl of MTT solution at 5mg/ml was added and incubated for 4 h. Dimethyl sulfoxide (DMSO) in volume of 100 µl is added into each well to dissolve the purple formazan formed. The colorimetric assay is measured and recorded at absorbance of 570 nm using a plate reader (EXL 800 , USA). The relative cell viability in percentage was calculated as (A570 of treated samples/A570 of untreated sample) X 100.

Conc.(μM)	WI38	WISH	HePG2	MCF7	HCT116	PC3	MDA-231	Caco-2	Hela
DOX									
100	7.8	4.7	6.3	6.2	7.1	8.8	4.1	15.1	7.3
50	14.5	17.9	11.2	10.9	13.9	16.3	11.8	24.7	12.1
25	21.2	26.8	14.1	14.3	18.7	21.7	16.2	36.8	18.9
12.5	34.0	32.6	28.3	26.9	31.4	38.9	27.3	42.6	30.8
6.25	50.3	61.4	45.8	41.5	47.9	59.2	36.1	62.0	51.7
3.125	64.9	75.4	57.6	58.4	60.5	73.6	52.6	78.3	62.4
1.56	87.4	89.7	71.2	69.1	73.8	95.3	61.7	100	74.0
Aotaphenazine (A-1)									
100	44.3	49.4	35.0	29.6	27.9	36.1	26.9	38.5	29.1
50	56.4	60.3	49.3	41.2	42.3	48.3	37.4	53.4	44.6
25	70.7	72.9	56.7	52.9	56.4	63.5	50.8	64.9	59.3
12.5	83.9	85.2	69.6	65.4	71.8	75.7	58.6	70.7	64.4
6.25	97.5	98.7	83.9	78.5	83.7	96.1	75.5	92.4	95.2
3.125	100	100	98.5	98.3	100	100	93.9	100	100
1.56	100	100	100	100	100	100	100	100	100

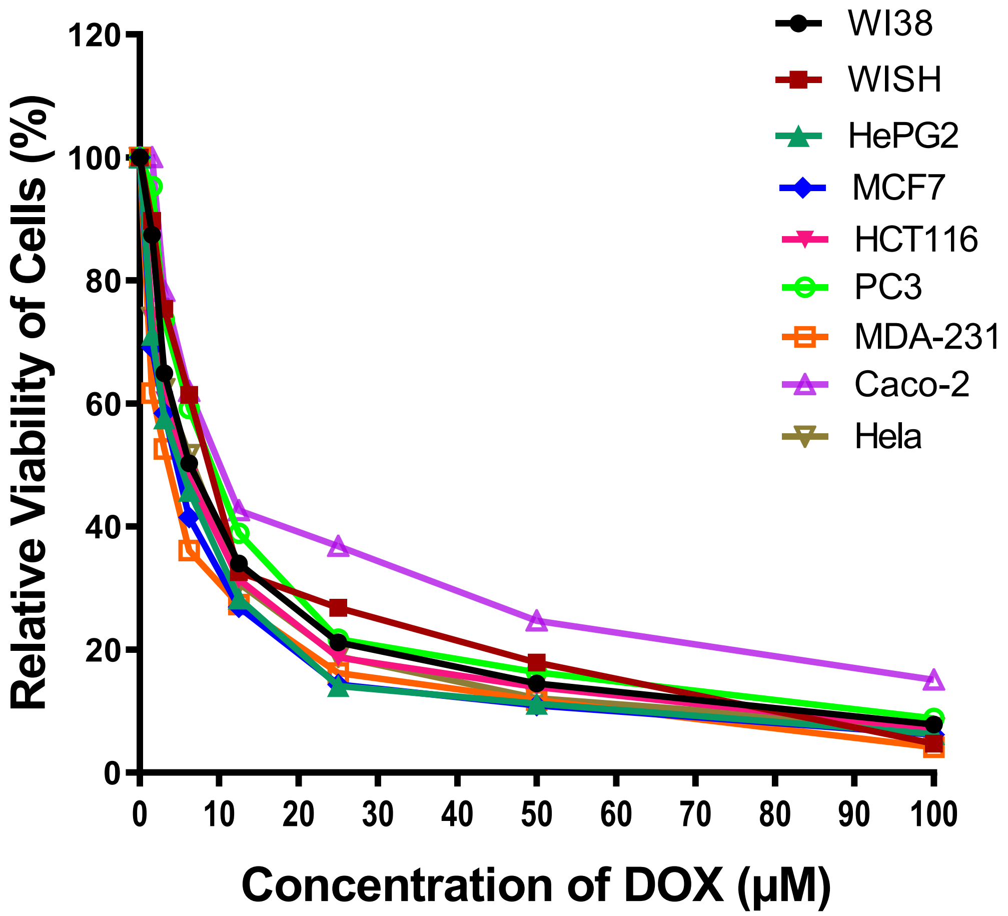
Relative Viability of Cells (%)
120
100
80
60
40
20
0
0
10
20
30
40
50
60
70
80
90
100
Concentration of DOX (μM)
WI38
WISH
HePG2
MCF7
HCT116
PC3
MDA-231
Caco-2
Hela

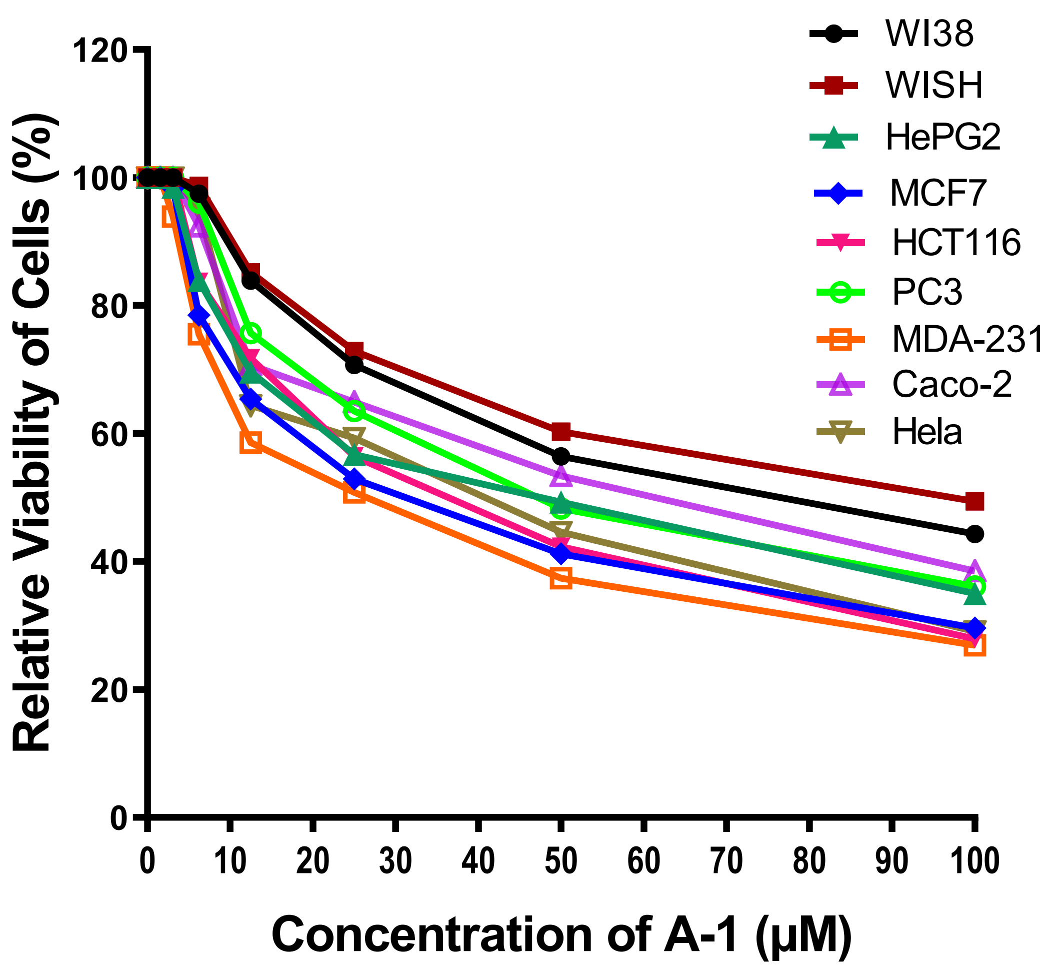

Relative Viability of Cells (%)
120
100
80
60
40
20
0
0
10
20
30
40
50
60
70
80
90
100
Concentration of A-1 (μM)
WI38
WISH
HePG2
MCF7
HCT116
PC3
MDA-231
Caco-2
Hela

8. Flow Cytometry

- **Cell Cycle Distribution Analysis**

The antiproliferative effects of aotaphenazine on MDA-MB-231 cells were quantitatively assessed through cell cycle analysis using propidium iodide (PI) DNA staining. Following treatment with IC50 concentrations (determined from prior MTT assays) for 48 hour time points, approximately 1×10^6 cells per condition were harvested, washed with cold PBS, and fixed in 70% ice-cold ethanol at -20°C for a minimum of 12 hours to ensure complete permeabilization. Fixed cells were subsequently treated with a staining solution containing 50 µg/mL propidium iodide (Sigma-Aldrich), 100 µg/mL RNase A (Thermo Fisher), and 0.1% Triton X-100 in PBS for 30 minutes at 37°C in the dark. Cell cycle profiling was performed on a BD FACSAria III flow cytometer (BD Biosciences) equipped with a 488 nm laser, with fluorescence emission collected through a 617/25 nm bandpass filter. For each sample, a minimum of 20,000 single-cell events were acquired, with doublet discrimination achieved through pulse-width versus pulse-area gating. The resulting DNA content histograms were analyzed using ModFit LT 5.0 software (Verity Software House), which applied the Dean-Jett-Fox model to quantify the percentage of cells in G0/G1 (diploid DNA content), S (DNA synthesis), and G2/M (tetraploid DNA content) phases, with additional quantification of sub-G1 populations indicative of apoptotic cells.

- **Apoptosis Induction Assessment**

The apoptotic response to aotaphenazine treatment was rigorously evaluated through Annexin V-FITC/propidium iodide (PI) dual staining, following established protocols from the BD Pharmingen Annexin V-FITC Apoptosis Detection Kit. Briefly, 5×10^5 cells were collected at each time point, gently washed twice with cold PBS, and resuspended in 100 µL of 1× binding buffer containing 5 µL Annexin V-FITC and 5 µL PI (50 µg/mL final concentration). After 15 minutes of incubation at room

temperature in the dark, 400 μL of additional binding buffer was added immediately prior to analysis. The flow cytometer was calibrated daily using BD CompBeads to ensure proper fluorescence compensation, with single-stained controls included in each experiment to establish compensation matrices. Quadrant analysis was performed using FACSDiva 8.0.1 software to distinguish four distinct populations: viable cells (Annexin V-FITC negative/PI negative, lower left quadrant), early apoptotic cells (Annexin V-FITC positive/PI negative, lower right quadrant), late apoptotic cells (Annexin V-FITC positive/PI positive, upper right quadrant), and necrotic cells (Annexin V-FITC negative/PI positive, upper left quadrant). Fluorescence-minus-one (FMO) controls were included to validate gating strategies and ensure accurate population discrimination.
