# Supplementary material for: Aotaphenazine, a rare hydrophenazine, targets topoisomerase II with anticancer efficacy: In silico to in vitro evidence
Source: PLoS One. 2025 Dec 5;20(12):e0338135. doi: 10.1371/journal.pone.0338135 (PMC12680189; doi:10.1371/journal.pone.0338135)
Supplement: Attachment — Submitted filename: Response.docx [file pone.0338135.s002.docx]

Dear editors,

Firstly, the authors would like to sincerely thank the Editor and Reviewers for their valuable time, constructive feedback, and insightful comments. Their suggestions have greatly contributed to improving the clarity, rigor, and overall quality of our manuscript. We have carefully revised the paper in accordance with all recommendations as detailed below:

Reviewer #1: Dear Author,

I have gone through the manuscript, it is fine in majority of aspects. There are few aspects that needs attention like:

1. The experimental section needs to be more elaborate with proper references.

Response: We thank the reviewer for this valuable comment. The Experimental Section has been expanded with detailed methodological descriptions and appropriate literature references.

2. In the references, the authors may provide full details of the authors instead of mentioning et al.
Response: We thank the reviewer for this helpful suggestion. The reference list has been revised to include the full author names for all citations, replacing et al. where applicable, in accordance with the journal’s referencing guidelines
3. Under conclusions the details of in vitro studies should be clearly discussed and it should be brief.
Response: We appreciate the reviewer’s helpful comment. The conclusion has been revised to briefly summarize the *in vitro* findings, including Topo II inhibition, cytotoxicity in cancer and normal cells, and flow cytometry results, providing a clearer and more focused summary.
4. The legends to the figures and tables should be more elaborate and self explanatory.

Response: We sincerely thank the reviewer for this valuable suggestion. All figure and table legends have been thoroughly revised to include detailed experimental descriptions, key parameters, and concise interpretations

5. Under cell cycle and apoptosis assay, only Aotaphenazine treated MDA-MB-231 cells are shown. Why the control cell data is not shown in the figure.

Response: We thank the reviewer for this important observation. In the revised version, the control (untreated) MDA-MB-231 cell data have now been included alongside the aotaphenazine-treated samples in both the cell cycle and apoptosis figures. This addition allows for a clear visual comparison between treated and control groups.

Reviewer #2: Reviewer Comments
Give the citation reference for the characterization of Aotaphenazine’s chemical structure, like 1HNMR/13CNMR & MS analysis.

Response: We thank the reviewer for this valuable suggestion. The reference detailing the full spectroscopic characterization of aotaphenazine (¹H NMR, ¹³C NMR, and MS data) has now been cited in the revised manuscript as Reference [38], which reports its isolation and structural elucidation by Prof. Masami Ishibashi and co-workers. Notably, one of the co-authors of that study, Dr. Mohamed S. Abdelfattah, who was the first author of the isolation paper, is also a co-author of the present manuscript.
Fig. 2: Include the two-dimensional pictures for each reference DOXO and Aotaphenazine using the DNA-Topo II complex, supported with each 3D graphic to better explain the many types of amino acid interactions.

Response: We thank the reviewer for this excellent suggestion. The revised Figure 2 now includes both the (2D) and (3D) interaction diagrams for doxorubicin (DOXO) and aotaphenazine bound to the DNA–Topo II complex. The updated panels clearly illustrate hydrogen bonding, π–π stacking, and hydrophobic interactions with key amino acid residues and nucleobases, providing a more comprehensive visualization of the binding modes.

Fig. 7: Images C1, C2, and C3 require further clarification.

Response: We thank the reviewer for this helpful observation. The legend and figure description for Figure 7 have been revised to provide clearer explanations of images C1, C2, and C3
